# Efficiency of a Combination of Polyethylene Glycol and Psyllium on the Evacuation of Sand Compared to Established Treatment Regimes in Sand Enteropathy of the Horse

**DOI:** 10.3390/ani16162507

**Published:** 2026-08-12

**Authors:** Lena Karlotta Lousberg, Roswitha Merle, Ann Kristin Barton

**Affiliations:** 1 Equine Clinic Hochmoor, 48712 Gescher, Germany; 2 Veterinary Department, Freie Universitaet Berlin, 14163 Berlin, Germany; 3 Institute of Veterinary Epidemiology and Biostatistics, Freie Universitaet Berlin, 14163 Berlin, Germany

**Keywords:** horse, sand enteropathy, psyllium, polyethylene glycol, Macrogol, paraffin oil, laxative, sodium sulfate

## Abstract

The accumulation of sand in the abdomen is a rising risk for colic in horses. Sand mainly accumulates in the ascending colon in the cranioventral abdomen. Evacuation of sand is facilitated by the application of psyllium supported with a laxative via nasogastric intubation. Laxatives currently used are either sodium sulfate/magnesium sulfate or mineral oil. The aim of this study was to compare the efficiency of polyethylene glycol, a laxative already used in human medicine for chronic constipation, with that of the above-described, established therapy options. Therefore, 44 horses diagnosed with sand enteropathy were randomly assigned and received psyllium in combination with either sodium sulfate, mineral oil or polyethylene glycol for four days. Pre- and post-therapy radiographs were assessed using a modified scoring system of Keppie et al. and Kendall et al. Adverse side effects during treatment were recorded and compared between treatment options. Median score reduction of sodium sulfate was −2 points, of polyethylene glycol −3.5 points and of mineral oil −2.5 points. Sand severity score was significantly reduced following all treatment options (*p* < 0.001), but did not differ between groups (*p* = 0.428). As all therapy regimens were successful in eliminating sand, polypropylene glycol might be the best option, as no adverse effects have been described in the literature or were seen in this study.

## Simple Summary

The accumulation of sand in the large colon can cause an abundance of gastrointestinal clinical signs and is regarded a rising cause of acute and recurrent colic in horses. Therapy aims at the evacuation of sand from the intestine. This is commonly achieved by daily administration of psyllium supported with a laxative by nasogastric intubation. Until now, laxatives described in the literature are either sodium/magnesium sulfate, a salt that increases the osmotic gradient, pulling water into the intestine, or mineral oil, which covers the intestinal tract and feces, preventing the absorption of water and lubricating the feces. Both laxatives are known to occasionally have severe side effects such as diarrhea, mild colic, malabsorption and other intestinal disturbances. This study evaluates the efficacy and practicability of polyethylene glycol, already successfully applied in human medicine, as a new laxative in comparison to already established methods.

## 1. Introduction

Sand enteropathy makes up to 5–11% of referral colic cases worldwide [1,2,3]. The literature describes an increase in clinical cases of sand enteropathy over the past ten years due to the uprise of extensive management practices [4]. Accumulation of sand in the abdomen has several possible causes, subdivided into voluntary and involuntary uptake. Geophagia is considered to be caused by boredom or mineral deficits [5]. The latter is a consequence of certain management practices such as feeding from the ground, or low daily herbage allowance combined with certain soil characteristics found in arid geographic areas [6,7].

Sand commonly sediments in the large colon [8,9,10], as slower intestinal borborygmi allow heavy particles to accumulate in the ventral portions [9]. In the intestine, the sand can cause mucosal irritation, obstruction of the lumen or decreased motility [11,12,13].

The clinical presentation of horses with sand enteropathy can vary from acute to recurrent colic, diarrhea, fever, weight loss, inappetence and poor performance [1,3,4,12,13,14,15,16]. There are many diagnostic tools described in the literature such as rectal palpation [1,9,17], fecal glove sedimentation test [6,18], sonography [10] and auscultation of the ventral abdomen [17]. Abdominal radiography continues to be considered the gold standard for diagnosis of sand enteropathies [9,17]. Three to four latero-lateral images of the ventral abdomen are taken [1,9,19,20]. Sand appears as a radiopaque mass [9,20]. Radiographic imaging can be rated by different graduation systems [9,20,21]. In the literature, the preferred scoring system is by Keppie et al. [4,9].

Therapy aims at the evacuation of sand from the intestine [17], which is thought to be achieved by hydrocolloid produced by psyllium [22]. Two studies showed efficacy of psyllium in the clearance of sand [14,15]. This effect was more evident when Psyllium was given via nasogastric intubation rather than through feed [14]. In recent years, several studies evaluating the effect of psyllium alone, compared to combinations of psyllium and a laxative, found the combinations to be more effective [14,23,24]. Laxatives used in these studies were magnesium sulfate at 1 g/kg BW sid [14,23] and mineral oil at 2 L per patient per day [24]. A combination of psyllium and sodium sulfate (50–250 g/horse) for the evacuation of sand was described in a study from Switzerland [4].

Side effects have been reported using laxatives in horses. Sodium sulfate (SS) and magnesium sulfate are osmotic laxatives [25]. They retain water in the intestinal lumen. As side effects of these saline laxatives mild colic-like pain [25], secondary gastric dilatation in horses with ileus [25] and severe water and electrolyte imbalances [26] are described. There are case reports on the occurrence of magnesium toxicosis due to the application of magnesium sulfate [27], causing clinical signs like tremors, recumbency and paralysis. An antagonization with intravenous calcium solution was described to be successful [27].

Mineral oil (MO) works as a lubricant and mild laxative [25]. Since it covers the intestinal mucosa and cannot be resorbed, it can cause malabsorption of both water-soluble and fat-soluble substances, such as salts or vitamins [25]. Fatal outcomes have been reported following inadvertent intubation of the trachea causing iatrogenic aspiration pneumonia [28].

Polyethylene glycol (PEG) is a linear polymer. Although it has been used in human medicine for children as well as adults with chronic constipation to support bowel emptying for many years [29,30,31], PEG has only recently been introduced to be used in equine medicine. It is an osmotic laxative [30] and its mode of action is building hydrogen connections, thus retaining water in the intestinal lumen [30]. In human medicine, observed adverse effects include diarrhea, loose stool, flatulence and nausea [30,32]; however, no changes in blood chemistry appear [32]. In a long-term study adverse effects were classified as mild to moderate and PEG was considered safe and effective [32]. The dose of 0.24 g/kg BW is considered to be safe and effective and was therefore adapted for this study [33,34,35]. For this reason we decided to adopt this dosage for our study protocol.

The aim of our study was to test the efficacy of polyethylene glycol in comparison to sodium sulfate and mineral oil in treatment regimens with psyllium. We hypothesized that efficacy to reduce sand accumulation is not significantly different between the three treatment regimes of sodium sulfate, mineral oil and polyethylene glycol.

## 2. Materials and Methods

### 2.1. Study Population

This prospective study included 44 horses treated for moderate–severe sand enteropathy at a private equine clinic in Western Germany. All horses received a routine clinical work-up including clinical examination, clinical pathology (RBC, WBC, total protein, leucocytes, blood gas analysis), rectal examination, sonography and radiography of the abdomen.

### 2.2. Radiography and Classification of Sand Enteropathy

Horses were diagnosed with moderate–severe sand enteropathy by abdominal radiography using a stationary X-ray machine (Spellmann/Bochum Editor hfe 501, EBA AG, Ansbach, Germany), the image processing system Gierth scope touch (GIERTH X-Ray international GmbH, Riesa, Germany), and a Canon CXDI-810C wireless wall-mounted plate (Canon Medical Systems GmbH, Neuss, Germany). Latero-lateral images were acquired using 100 kV, 250 mA and 250–320 ms. Two to three radiographs of the ventral abdomen were taken, depending on the horse’s size and the dorsal border of the sand accumulation. If possible, the largest coherent accumulation was depicted in one frame to be used for future reference. The time of acquisition was used as reference time (t0).

A scoring system was developed to describe radiopacities in the abdomen consistent with sand accumulation according to previously published systems. Location, number, opacity and homogeneity of the accumulation were scored using the system published by Keppie et al. [9]. Location was divided into two characteristics: cranioventral (=1 points) and other (=0 points). Number was divided into three characteristics: 0 accumulations (=0 points), 1–2 accumulations (=1 point) and 3 or more accumulations (=3 points). Homogeneity was divided into three characteristics: heterogeneous (=0 points), mixed (=1 point) and homogeneous (=2 points). Opacity was divided into three characteristics: less opaque than a rib (=0 points), mixed (=1 point) and more opaque than a rib (=2 points). The size of the accumulation was scored using Kendall et al. [20], as visualization of the ventral aspect of the ribs was not possible in every patient, which was required for scoring the size using Keppie’s system [9]. Scores were given according to length and width measurement as shown in Table 1. No accumulation was scored 0 points. An accumulation smaller than 5 × 5 cm was scored 1 point, over 5 × 5 cm 2 points, smaller than 5 × 15 cm/15 × 5 cm 2 points, smaller than 5 × 15 cm/15 × 5 cm but close to the ventral abdominal wall 3 points and over 5 × 15/15 × 5 cm 4 points. The overall score was calculated by adding the reached score of each category. A maximum score of 12 could be reached. Horses with ≥7 points were included in the study, for which acquisition of radiographs post therapy followed. Radiographs were scored twice by two observers, a young veterinarian (LL) and an experienced clinician (AKB).

### 2.3. Therapy of Sand Enteropathy

On the day of diagnosis, horses were randomly allocated to one of three treatment groups by drawing lots with the exception of 6 horses, in which treatment had been started prior to diagnosis. In this case, horses were left with the treatment begun. The three groups were defined as followed: (1) psyllium (1 g/kg BW) and SS (0.5 g/kg BW, Dr. Lohmann Diaclean GmbH, Dortmund, Germany), (2) psyllium (1 g/kg BW) and MO (5 mL/kg BW, Paraffinum Perliquidum WDT, Garbsen, Germany) or (3) psyllium (1 g/kg BW) and PEG (0.24 g/kg BW, Macrogol AL—Aluid Pharma, Laichingen, Germany). Psyllium and laxative were administered within water (3–6 mL/kg BW) via nasogastric intubation once a day over a course of 4 days. During the treatment period, horses were kept on boxrest in a stable with wood shavings. Hay was supplied twice daily to horses without ongoing abdominal discomfort. Horses observed ingesting wood shavings received a muzzle during fasting periods. Horses exhibiting signs of colic received analgesics, such as metamizol (40 mg/kg BW) and butylscopolamine (0.2 mg/kg BW) intravenously according to pain levels.

### 2.4. Post-Treatment Radiographs

On the fifth day (t1), a second set of abdominal radiographs was taken to assess the results of treatment. These radiographs were taken and scored in the same manner as on the initial consultation. Accumulations scoring 4 points and lower were classified as resolved, while score reductions of >2 points were defined as improvement. If the accumulation had not resolved by that time, the owners were given the choice to either continue treatment via nasogastric intubation or to feed psyllium at home. Those results were not included in this study.

### 2.5. Statistics

Clinical data were recorded in a digital patient documentation system (easyVET™, VetZ Gmbh, Isernhagen, Germany) and Microsoft Excel™. IBM SPSS Statistics 29.0.1. was used for the descriptive evaluation of the data, as well as statistical analysis and chart creation. A *p*-value of *p* < 0.05 was considered significant. Continuous data were assessed visually for normality. The median value of the four different scorings of observers 1 and 2 was used for calculations. Treatment efficiency was calculated by subtraction of the X-ray score at t1 from the score at t0. The scores at t0 and t1 were compared using the Wilcoxon signed-rank test. Differences in efficacy between the treatment groups were tested using the Kruskal–Wallis test. Patients were grouped by severity of the accumulation at t0, defining patients scoring 7–9 points at initial exam as moderate and 10–12 points as severe. Treatment efficiency was compared between the two groups using the Mann–Whitney U-test.

Intra- and inter-observer reliability was determined for verification of the radiographic score. They were assessed using the intraclass correlation coefficient ICC between the two measurements of scorer 1 and between the calculated median scores of scorer 1 and 2. Benchmarks applied according to Koo and Li [35] were: <0.5 indicating poor reliability, 0.5–0.75 indicating moderate reliability, 0.75–0.9 indicating good reliability, and >0.9 indicating excellent reliability.

## 3. Results

### 3.1. Study Population

The study population consisted of 44 horses submitted to a private clinic in Western Germany between October 2024 and May 2026. It included 26 mares, 15 geldings and 3 stallions of various breeds: Shetland Pony (9), Westphalian Horse (6), Icelandic Horse (4), Irish Tinker (3), Pony (2), German Riding Pony (2), Warmblood (2), Haflinger (2), Andalusian (2), Fjord horse (2), KWPN (2), Quarter horse (1), Hanoverian (1), Hungarian Sporthorse (1), German Sporthorse (1), Friesian-mix (1), Friesian (1), Oldenburger (1), and Thoroughbred (1). Ages ranged from 0.5 years to 30 years, with a mean age of 8 years. The horses’ weights ranged from 81 kg to 672 kg (mean 381 kg). Management information was available for 29 horses: 14 were kept in stables with paddock access, 5 in open stables, and 10 had pasture access.

Reasons for admission to the clinic were acute colic (37), weight loss (1), pyrexia (1) and other reasons (5). From 37 horses admitted with signs of colic, rectal examination revealed meteorism in 17 horses and impaction in 13 horses. No abnormalities were detected or rectal examination was not possible in 14 horses.

### 3.2. Treatment

All horses were treated with psyllium adding differing laxatives: 14 horses were treated with SS, 16 with MO and 14 with PEG. Two horses of the SS group, three of the MO group and one of the PEG group received treatment via nasogastric intubation by the referring veterinary surgeon or at the clinic prior to diagnosis and were left in that group, so treatment was not randomly assigned in these.

In one horse of the SS group, treatment had to be stopped on day two as it showed insufficient gastric emptying. One horse of the SS group developed diarrhea but therapy was continued. One horse of the PEG group underwent laparotomy but showed persistent signs of colic and ultrasonographic abnormalities despite radiographic evidence of successful clearance from sand. During surgery, right dorsal displacement was diagnosed and corrected. Ten out of 44 horses experienced at least one or more episode of colic during therapy while admitted to the hospital (3/14 SS, 5/16MO, 2/14 PEG group).

### 3.3. Radiography

Overall median radiographic score at the time of diagnosis (t0) was 10 (ranging from 7 to 12 points). In the SS group, X-ray scores at t0 ranged from 7 to 12 (median 10), in the MO group from 8 to 12 (median 10) and in the PEG group from 7 to 10 (median 9.25).

Following four days of therapy, overall median X-ray score t1 was 7.5 points (0–12 point range). In the SS group, the scores ranged from 4 to 10 points (median 8), in the MO group from 0 to 12 points (median 7.5 points), and in the PEG group from 0 to 10 points (median 6.5).

**Figure 1:**
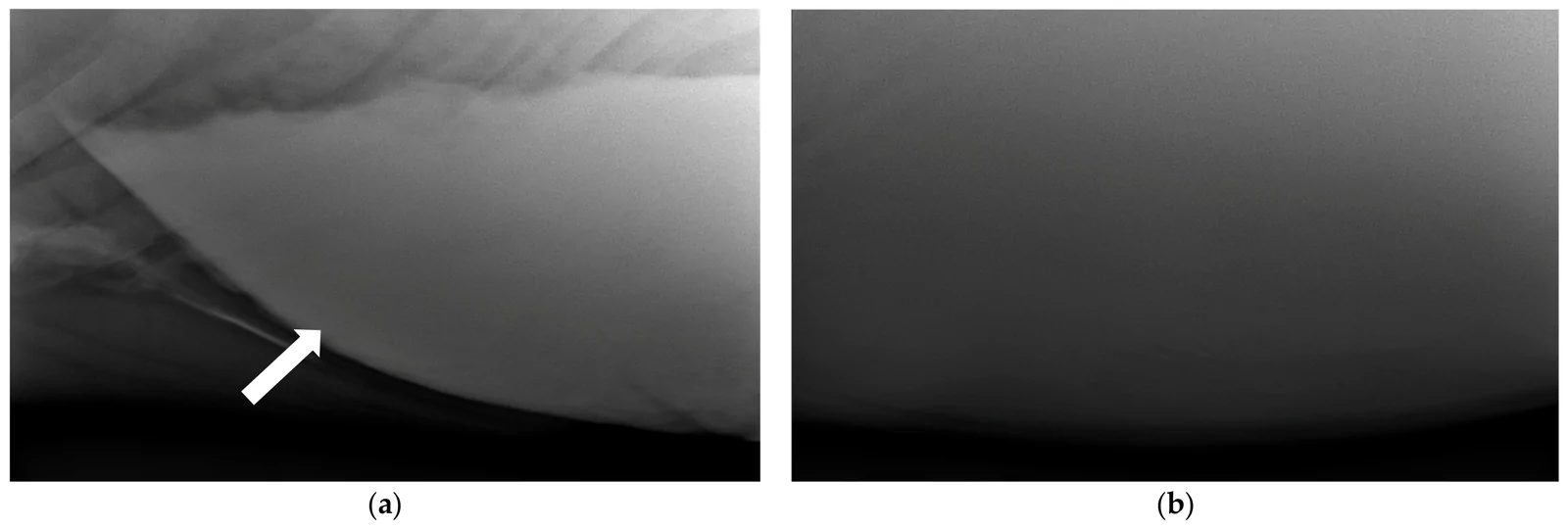
Latero-lateral radiographs of the abdomen in the same patient acquired at two different time points t0 and t1; cranial is to the left (**a**) sand accumulation (arrow) with an X-ray score of 10/12 points at the time of diagnosis (t0), and (**b**) with an X-ray score of 0/12 points post treatment (t1).

Therapy resulted in changes in radiographic scores ranging from −12 to +2 points with a median change of −2 points. In the SS group, median X-ray score reductions ranged from −4 to 0 points (median −2 points), in the MO group from −12 to +2 points (median −2.5), and in the PEG group from −9 to +1 points (median −3.5). An example is shown in Figure 1. 

Using the Wilcoxon signed rank test, radiographic scores were significantly lower at t1 than t0 across all groups with a median reduction of −2 points (*p* < 0.01). Overall, therapy was associated with a significant reduction in sand accumulation.

**Figure 2:**
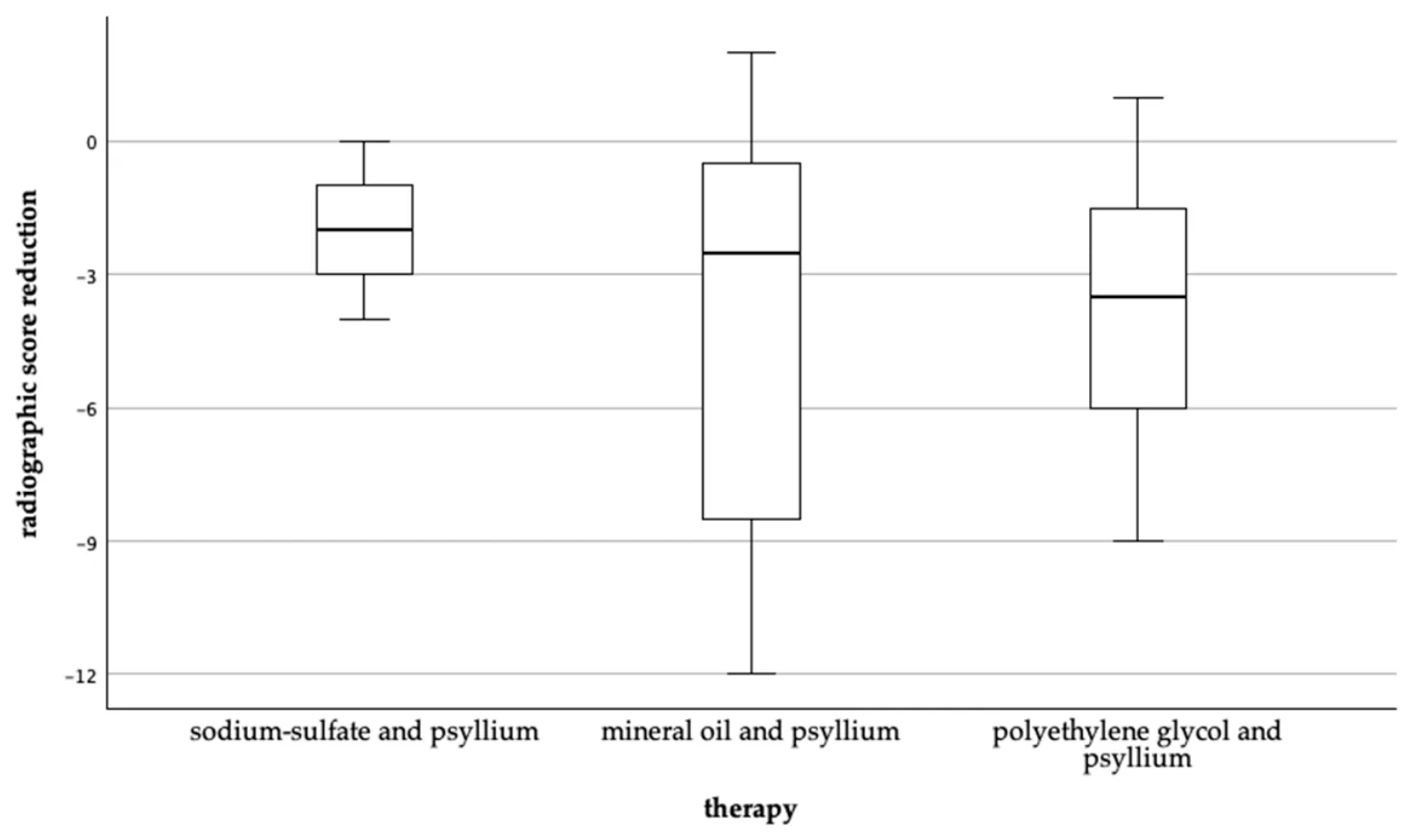
Boxplot of score reduction between treatment regimes.

Using the Kruskal–Wallis test, radiographic score reductions were not significantly different between treatment groups (*p* = 0.428), showing similar clearance of the PEG treatment to the established treatment groups SS and MO (Figure 2).

Eleven out of 44 horses reached X-ray scores of 4 and lower after 4 days of therapy and could thus be classified as fully cleared. Twenty-seven out of 44 horses did improve the radiographic score by at least 2 points. Nine out of 44 animals showed no radiographic score reduction after therapy.

Using the Mann–Whitney U-test, no statistically significant difference (*p* = 0.941) in treatment efficiency between the two degrees of severity (moderate and severe) was displayed.

The ICC for the intra-observer reliability was 0.975 (confidence interval 0.962–0.984) for observer 1 and 0.995 (confidence interval 0.992–0.997) for observer 2, indicating an excellent reliability of the radiographic score. The ICC for inter-observer reliability was 0.993 (confidence interval: 0.987–0.996), indicating excellent inter-observer reliability of the radiographic score.

## 4. Discussion

Our study suggests that polyethylene glycol provides comparable efficacy to the established treatment regimens reported in the literature.

### 4.1. Radiographic Score

The scoring system used in this study, adapted from Keppie et al. [9] and Kendall et al. [20], was originally developed to grade sand accumulations according to their clinical relevance by establishing cut-off values. The proposed values were 7 and 2 points, respectively. We chose to primarily apply Keppie et al.’s scoring system because it provided a more versatile approach for assessing the severity of sand accumulations. Consistent with the difficulties described by Kendall et al., the ventral aspect of the ribs could not be visualized in many horses. Therefore, the scoring system described by Kendall et al. was used to assess the size of sand accumulation.

The new composed radiographic score showed excellent intra- and inter-observer reliability. However, it was the first time being applied to assess the severity of sand accumulations. Moreover, reliability testing only contains gradings of two observers at two time points. For further verification the score should be applied and tested in other studies.

During the study, a large proportion of horses scoring 10 points made the study population appear very equal. This, however, did not agree with our subjective assessment. Considerable differences in the size of sand accumulations scoring 10 points were not reflected by the scoring system. Accumulations ranged in size from 5 × 15 cm to up to 23.5 × 35 cm. Consequently, achieving a reduction in the size category was considerably more difficult for horses with very large accumulations than for those with smaller accumulations despite having the same score. This limitation was not mentioned before and may reflect the larger sand accumulations encountered in this study compared to previous reports (20). We anticipated that this aspect would be partly compensated by including additional scoring criteria beyond the size alone.

Additionally, the scoring method used did rate three or more accumulations higher than one or two, suggesting more severe disease. This raises the question whether more accumulations always equal a more severe disease or if one or two large accumulations should be classified as more severe.

In a subset of horses, an increase in the X-ray score was observed. This is physically not thought to be possible as the patient had no access to sand. This observation could be explained by one large accumulation separating into several smaller ones during therapy and therefore scoring higher. It is therefore questionable whether several smaller accumulations should always receive a higher score reflecting more severe clinical pathology.

Despite these shortcomings in grading severity, a new benefit of radiographic scoring could be the prediction of therapy duration and therapy success. However, a study comparing accumulation score and therapy efficacy using only one of the established treatment regimes would be necessary and requires a more detailed classification of the accumulations.

### 4.2. Alternative Monitoring Methods

Radiography of the abdomen requires relatively high exposure settings. In the present study, many horses had persistent sand accumulation after 4 days of treatment, necessitating a third or even fourth radiographic examination. This not only prolongs hospitalization or may require re-admission for repeated radiography, but also increases cumulative radiation exposure for staff members. Therefore, alternative methods for monitoring treatment response would be useful. In the current literature several diagnostic approaches such as fecal glove sedimentation test or ultrasonography are described. These could be considered to determine the most suitable time point for repeat radiographic examination. Criteria, such as no further sand excretion or resolution of hyperechogenic filling of the colon visible on ultrasound, could be applied. These methods, however, do have limited sensitivity and specificity [10,18], making it difficult to determine the correct time point in every patient. A future prospective study would be a great advancement.

### 4.3. Laxatives

No severe side effects were observed in any of the treatments. This could relate to the short period of administration, the high level of precaution taken or the small study groups. One horse in the sodium sulfate group developed mild diarrhea during the course of the four-day treatment period but the treatment could be continued. Due to the risk of severe diarrhea or colitis, treatment was not further extended beyond the four days and the horse was discharged with psyllium treatment only to be administered over the feed for several more weeks.

According to our results, mineral oil shows a tendency towards the most efficient reduction/clearance of the sand accumulation, although not statistically significant. However, severe adverse effects have been described in the previous literature [25]. The most serious side effect is lethal pneumonia due to the aspiration of mineral oil during incorrect nasogastric intubation [28]. For therapy of sand enteropathy, mineral oil needs to be administered over the course of at least four days, posing a risk for aspiration pneumonia [24]. No cases of aspiration pneumonia following application of saline laxatives have been reported to the authors’ knowledge. This could be explained by the easier flush of the nasogastric tube before removal when using a water-soluble laxative. A high level of precaution is essential to safely administer mineral oil. Therefore, its use should be evaluated for each individual patient. Especially in those horses reacting very defensively to nasogastric intubation, the safer option of the three laxatives should be chosen.

Polyethylene glycol showed a good degree of solution, especially in cold water, which is favorable for psyllium administration, since swelling before administration is delayed. We observed no adverse effects, which could be due to the small group size; in a larger study population, adverse effects might occur. At the moment, there is no pharmaceutical approval for PEG in veterinary use. Unfortunately, our study is only the second study describing the use and safety of polyethylene glycol for horses, although it is commonly used at clinics all over Europe. Further scientific proof for its applicability for impaction colic would be very interesting.

### 4.4. Treatment Efficacy

The present study was not designed or powered to perform inferiority testing. Thus, the non-statistical results of this study can only be regarded as a hint that the different treatments might not differ in their efficacy of clearing sand from the abdomen. Therefore, we recommend larger prospective studies using the same treatment protocols and to perform these studies in geographically different regions.

Thirty-three of 44 horses did not achieve full clearance from sand after 4 days of treatment. We did see lower clearance rates than in earlier studies [23,24,36]. A possible reason might be the different radiographic score, the change to sodium sulfate or a regional influence changing the composition of the accumulated sand. It could also reflect insufficiency of treatment or insufficient treatment duration. Most horses would have required prolonged treatment, but owner compliance was difficult due to rising medical costs. Additionally, there are safety concerns with laxative treatments over prolonged time periods as discussed above [25,37], although they did not show in this study with only 4 days of treatment. Therefore, having the possibility to continue treatment of these horses at home feeding PGE and psyllium, safety concerns could be minimized and medical costs reduced. The initial treatment with mineral oil and psyllium via nasogastric intubation followed by a period of feeding PEG and psyllium at home could be an option for long-term treatment.

Nine out of 44 horses did not show any decrease in X-ray score at all, and 44% of these horses were Shetland Ponies. This is in accordance with Entwistle’s observations that miniature breeds are more likely to be non-responders [15]. Shetland Ponies were also the largest group in our study (9/44). The findings in this study highlight the importance and necessity of future work assisting treatment options for this particular patient group. A possible explanation for the poor treatment response could be the smaller amount of water being administered due to the patient’s size. Also, the smaller diameter of the nasogastric tube used for this breed could be a cause, as obstruction is more common. A possible treatment regimen could be the administration of psyllium twice daily to achieve a higher water and laxative intake over two intubations rather than a single treatment.

### 4.5. Prevention

Owner compliance for management optimization to prevent recurrence of sand enteropathy can be challenging. Two of 44 horses had recurrent colic due to sand enteropathy and took part in this study at two different time points. These were regarded as individual incidences for statistics. Implementation of management advice can be challenging for owners. Creating an environment for horses strictly prohibiting access to sand has potential welfare implications. Moreover, we should broaden our horizon to alternative ways of sand intake, e.g., by water supply. In addition to management optimization, prevention of sand enteropathies could be an important factor, such as the supplementation of roughage on empty pastures [38], the supplementation of mineral deficiencies [11] or the elevated supply of hay in hay racks. To fully incorporate a sufficient prophylaxis regime for susceptible patients, more research in the pathophysiology of this disease is needed.

### 4.6. Limitations of the Study

As in all clinical studies, the population of horses studied was not uniform and it was not possible to match controls of the same breed, age and environmental conditions not affected by colic to our study groups, which would have reduced pre-participation bias and would have increased the statistical power. In addition, the above-discussed problems with the radiographic scoring system may have led to misclassification of horses and inclusion of horses of differing disease severity. Both scoring systems, Keppie et al. and Kendall et al., were developed to identify sand accumulations of clinical relevance to the horse; both may not be sensitive enough to monitor treatment success or prognosis regarding further management of the horse when the sand accumulation had been excreted completely after 4 days.

## 5. Conclusions

Our study found no statistically significant difference in the reduction in sand between treatment regimes of polyethylene glycol and psyllium, sodium sulfate and psyllium, and mineral oil and psyllium over the course of 4 days. With a median score reduction of −3.5 points, it showed similar clearing to the established treatment regimes. Moreover, our study challenges the so-far recommended treatment regime including sodium sulfate and psyllium, as it was equally effective.

The results suggest that a scientific reevaluation of recommended treatment durations is essential and possibly should be increased, as it was not sufficient to fully clear the sand in most cases. In nine cases, four days of therapy could not reduce the score by even one point. This could be connected to an insufficient scoring scheme, especially for larger accumulations, or to insufficient treatment duration. Additionally, to treat horses for longer periods, a safe laxative for long-term use is needed.

Since polyethylene glycol is already approved for long-term use in human medicine and it has been shown to be effective for sand clearance, it is a very promising option for the future of therapy of sand enteropathy.

## Figures and Tables

**Table 1:** Combined X-ray score by Keppie et al. [9] and Kendall et al. [20] used in this study to score X-ray images.

Points	Size (cm)	Number	Location	Opacity	Homogeneity
0	No sand	0	Other	Less opaque	Heterogenous
1	<5 × 5	1–2	Cranioventral	Mixed	Mixed
2	<5 × 15/<15 × 5	-	-	More opaque	Homogeneous
3	<5 × 15/<15 × 5 On ventral abdominal wall	≥3	-	-	-
4	>5 × 15/>15 × 5	-	-	-	-

## Data Availability

Data are contained within the article or Supplementary Materials; further inquiries can be directed to the corresponding author.

## Supplementary Materials

The following supporting information can be downloaded at https://www.mdpi.com/article/10.3390/ani16162507/s1, Table S1: Radiographic scoring of participants.

## References

[1] Loschelder J., Gehlen H. (2017). Sand colic in the horse—Review and case examples. Pferdeheilkunde.

[2] Miserski F., Sattler T., Schusser G.F. (2023). Epidemiology of colic in horses, a prospective study of colic incidence density rate and mortality rate in middle Germany. Pferdeheilkunde.

[3] Maxwell J.A.L. (2003). The Surgical Management of Sand Impaction in a Miniature Horse. Aust. Vet. J..

[4] Graubner C., Waschk M., Aepli H., Stauffer S., Gerber V. (2017). Sand-Enteropathy in 35 Equids in Switzerland. Pferdeheilkunde.

[5] McGreevy P.D., Hawson L.A., Habermann T.C., Cattle S.R. (2001). Geophagia in Horses: A Short Note on 13 Cases. Appl. Anim. Behav. Sci..

[6] Husted L., Andersen M.S., Borggaard O.K., Houe H., Olsen S.N. (2005). Risk Factors for Faecal Sand Excretion in Icelandic Horses. Equine Vet. J..

[7] Jurjanz S., Collas C., Quish C., Younge B., Feidt C. (2021). Ingestion of Soil by Grazing Sport Horses. Animals.

[8] Ruohoniemi M., Kaikkonen R., Raekallio M., Luukkanen L. (2001). Abdominal Radiography in Monitoring the Resolution of Sand Accumulations from the Large Colon of Horses Treated Medically. Equine Vet. J..

[9] Keppie N.J., Rosenstein D.S., Holcombe S.J., Schott Ii H.C. (2008). Objective Radiographic Assessment of Abdominal Sand Accumulation in Horses. Vet. Radiol. Ultrasound.

[10] Korolainen R., Ruohoniemi M. (2002). Reliability of Ultrasonography Compared to Radiography in Revealing Intestinal Sand Accumulations in Horses. Equine Vet. J..

[11] Bertone J.J., Traub-Dargatz J.L., Wrigley R.W., Bennett D.G., Williams R.J. (1988). Diarrhea Associated with Sand in the Gastrointestinal Tract of Horses. J. Am. Vet. Med. Assoc..

[12] Kilcoyne I., Dechant J.E., Spier S.J., Spriet M., Nieto J.E. (2017). Clinical Findings and Management of 153 Horses with Large Colon Sand Accumulations. Vet. Surg..

[13] Hart K.A., Linnenkohl W., Mayer J.R., House A.M., Gold J.R., Giguère S. (2013). Medical Management of Sand Enteropathy in 62 Horses. Equine Vet. J..

[14] Kaikkonen R., Niinistö K., Lindholm T., Raekallio M. (2016). Comparison of Psyllium Feeding at Home and Nasogastric Intubation of Psyllium and Magnesium Sulfate in the Hospital as a Treatment for Naturally Occurring Colonic Sand (Geosediment) Accumulations in Horses: A Retrospective Study. Acta Vet. Scand..

[15] Entwisle I., McConnell E. (2025). Medical Treatment of Sand Enteropathy with Psyllium, Magnesium Sulphate and Paraffin Oil in 54 Western Australian Equids. Aust. Vet. J..

[16] Tharwat M., Sobayil F. (2025). Equine Colic: A Comprehensive Overview of the Sonographic Evaluation, Diagnostic Criteria, and Management of Different Categories. Open Vet. J..

[17] Niinistö K., Sykes B.W. (2022). Diagnosis and Management of Sand Enteropathy in the Horse. Equine Vet. Educ..

[18] Hukkinen V. (2015). The Diagnostic Accuracy of the Plastic Glove Test for Diagnosis of Sand Enteropathy in the Horse. Licenciate Thesis.

[19] Valli C. (2025). Diagnosis and Treatment of Equine Sand Enteropathy. UK-Vet. Equine.

[20] Kendall A., Ley C., Egenvall A., Bröjer J. (2008). Radiographic Parameters for Diagnosing Sand Colic in Horses. Acta Vet. Scand..

[21] Entwisle I., Byrne D., Lester G., McConnell E. (2025). Radiographic Area of Large Intestinal Sand Accumulation in Horses May Determine Clinical Significance. Aust. Vet. J..

[22] Mienaltowski M.J., Belt A., Henderson J.D., Boyd T.N., Marter N., Maga E.A., DePeters E.J. (2020). Psyllium Supplementation Is Associated with Changes in the Fecal Microbiota of Horses. BMC Res. Notes.

[23] Niinistö K., Hewetson M., Kaikkonen R., Sykes B.W., Raekallio M. (2014). Comparison of the Effects of Enteral Psyllium, Magnesium Sulphate and Their Combination for Removal of Sand from the Large Colon of Horses. Vet. J..

[24] Hotwagner K., Iben C. (2008). Evacuation of Sand from the Equine Intestine with Mineral Oil, with and without Psyllium. Anim. Physiol. Nutr..

[25] Gembicki N., Fey K. (2011). Laxatives in the horse—A review of the literature. Pferdeheilkunde.

[26] Lopes M.A.F., Moura G.S., Filho J.D. (1999). Treatment of Large Colon Impaction with Enteral Fluid Therapy. Proc. Am. Assoc. Equine Pract..

[27] Henninger R.W., Horst J. (1997). Magnesium Toxicosis in Two Horses. J. Am. Vet. Med. Assoc..

[28] Vainio K.M.E., Määttänen M.K., Mykkänen A.K., Huupponen A.K., Niinistö K.E. (2025). Iatrogenic Aspiration Pneumonia in Six Horses: A Retrospective Case Series. J. Equine Vet. Sci..

[29] Pashankar D.S., Bishop W.P. (2001). Efficacy and Optimal Dose of Daily Polyethylene Glycol 3350 for Treatment of Constipation and Encopresis in Children. J. Pediatr..

[30] Koppen I.J.N., Lammers L.A., Benninga M.A., Tabbers M.M. (2015). Management of Functional Constipation in Children: Therapy in Practice. Paediatr. Drugs.

[31] Chaussade S., Minić M. (2003). Comparison of Efficacy and Safety of Two Doses of Two Different Polyethylene Glycol-based Laxatives in the Treatment of Constipation. Aliment. Pharmacol. Ther..

[32] Di Palma J.A., Cleveland M.V., Mcgowan J., Herrera J.L. (2007). An Open-label Study of Chronic Polyethylene Glycol Laxative Use in Chronic Constipation. Aliment. Pharmacol. Ther..

[33] Becker R., Koch C., De Preux M. (2026). Surgical Removal of a Perirectal Melanoma through Sphincterotomy and Rectal Wall Resection in a Horse. Equine Vet. Educ..

[34] Somm F., Fürst A.E., Jackson M.A. (2026). Surgical Excision of Melanomas in the Anal Region and Reconstruction of the Anus in 11 Horses. Equine Vet. Educ..

[35] Koo T.K., Li M.Y. (2016). A Guideline of Selecting and Reporting Intraclass Correlation Coefficients for Reliability Research. J. Chiropr. Med..

[36] Niinistö K.E., Ruohoniemi M.O., Freccero F., Raekallio M.R. (2018). Investigation of the Treatment of Sand Accumulations in the Equine Large Colon with Psyllium and Magnesium Sulphate. Vet. J..

[37] Bäumer W., Emmerich I.U., Hamann M., Honscha W., Ibrahim C., Kaspar H., Kietzmann M., Kluge K., Meichner K., Moos M., Löscher W., Richter A., Potschka H. (2014). Pharmakotherapie bei Haus- und Nutztieren: Tierpflege in Forschung und Klinik.

[38] Hammock P.D., Freeman D.E., Baker G.J. (1998). Failure of Psyllium Mucilloid to Hasten Evacuation of Sand from the Equine Large Intestine. Vet. Surg..

