# Supplementary material for: Efficiency of a Combination of Polyethylene Glycol and Psyllium on the Evacuation of Sand Compared to Established Treatment Regimes in Sand Enteropathy of the Horse
Source: Animals (Basel). 2026 Aug 12;16(16):2507. doi: 10.3390/ani16162507 (PMC13508846; doi:10.3390/ani16162507)
Supplement: Supplementary file 1 [file animals-16-02507-s001.zip › Table S1 Radiographic scoring of participants.pdf]

breed	age	time of radiography	localisation	number of accumulations	opacity, compared to ventral part of a rib	homogeneity	score (from Keppie et. Al)	length (cm)	height (cm)	score (from Kendall et. Al)	overall score	group
Shetland pony	6	0	cranioventral and dorsal Colon	6	more opaque	homogeneous	8	24.703	7.994	4	12	MO
		1	cranioventral	3	more opaque	homogeneous	8	19.879	9.633	4	12	
Shetland pony	6	0	cranioventral + caudoventral	2	more opaque	homogeneous	6	27.456	10.84	4	10	MO
		1	Cranioventral + ventral	3	more opaque	homogeneous	8	34.308	10.733	4	12	
Quarter Horse	21	0	Cranioventral + ventral	2	more opaque	homogeneous	6	34.938	15.846	4	10	SS
		1	cranioventral	1	mixed	homogen	5	27.455	18.552	4	9	
irish Tinker	5	0	cranioventral+caudoventral+ dorsal	1	mixed	mixed	4	34.924	26.037	4	8	SS
		1		1	mixed	inhomogeneous	2	34.974	20.601	4	6	
Westphalian Horse	3	0	caudoventral	1	mixed	mixed	3	29.539	22.932	4	7	PEG
		1		0			0			0	0	
Shetland pony	18	0	Cranioventral + ventral	1	more opaque	homogeneous	6	34.923	21.321	4	10	SS
		1	Cranioventral + ventral	2	more opaque	homogeneous	6	32.955	21.484	4	10	
Shetland Pony	25	0	cranioventral	2	more opaque	homogeneous	6	25.797	13.535	4	10	MO
		1	ventral	1	more opaque	homogeneous	5	34.926	10.864	4	9	
Shetland pony	24	0	cranioventral + caudoventral	1	more opaque	mixed	5	36.316	15.557	4	9	PEG

		1	cranioventral + caudoventral	2	more opaque	mixed	5	33.731	15.868	4	9	
Icelandic horse	26	0	cranioventral	1	more opaque	homogeneous	6	24.017	9.952	4	10	MO
		1	cranioventral	3	mixed	mixed	6	28.48	10.813	4	10	
Westphalian Horse	15	0	ventral	2	more opaque	homogeneous	4	25.264	14.497	4	8	MO
		1					0	0	0	0	0	
Icelandic Horse	0.5	0	ventral	2	mixed	mixed	3	33.083	18.026	4	7	SS
		1	cranioventral + ventral	1	mixed	heterogeneous	3	34.065	7.984	4	7	
Hanoveranian	3	0	Cranioventral + ventral	2	mixed	mixed	4	35.114	13.517	4	8	SS
		1					0			0	0	
Pony	16	0	cranioventral	1	more opaque	mixed	5	27.779	16.019	4	9	SS
		1	Cranioventral + ventral	1	mixed	mixed	4	26.087	9.259	4	8	
Andalousian	6	0	Cranioventral + ventral	4	more opaque	homogeneous	8	34.983	9.006	4	12	MO
		1					0	0	0	0	0	
Shetland Pony	15	0	Cranioventral + ventral	1	more opaque	homogeneous	6	31.536	9.834	4	10	SS
		1	cranioventral	1	mixed	mixed	4	23.79	8.202	4	8	
Hungarian Sporthorse	9	0	ventral	2	more opaque	homogeneous	5	34.98	19.794	4	9	MO
		1					0	0	0	0	0	
Irish Tinker	6	0	Cranioventral + ventral	1	more opaque	mixed	5	34.996	23.505	4	9	PEG
		1	ventral+ caudoventral	1	mixed	mixed	3	12.503	20.42	4	7	
Pony	6	0	cranioventral	1	more opaque	homogeneous	6	32.136	16.911	4	10	MO
		1					0	0	0	0	0	
German Riding Pony	8	0	Cranioventral + ventral	3	more opaque	homogeneous	8	34.955	13.205	4	12	MO
		1	ventral	1	mixed	mixed	3	28.56	3.014	4	7	

Icelandic Horse	30	0	Cranioventral + ventral	1	more opaque	homogeneous	6	35.023	15.771	4	10	MO
		1	cranioventral + caudoventral	1	mixed	mixed	4	34.98	13.315	4	8	
Shetland Pony	25	0	Cranioventral + ventral	2	more opaque	homogeneous	6	27.727	11.394	4	10	SS
		1	ventral	1	mixed	mixed	3	25.775	11.806	4	7	
German Sporthorse	1.5	0	Cranioventral + ventral	1	more opaque	homogeneous	6	35	23.022	4	10	MO
		1	Cranioventral + ventral	1	more opaque	homogeneous	6	34.991	17.555	4	10	
Warmblood	2.5	0	Cranioventral + ventral	2	more opaque	mixed	5	34.957	23.485	4	9	PEG
		1	cranioventral	2	mixed	mixed	4	33.037	18.785	4	8	
Westphalian Horse	3	0	cranioventral	1	mixed	mixed	4	34.982	21.487	4	8	MO
		1	cranioventral	1	more opaque	homogeneous	6	30.708	14.662	4	10	
Westphalian Horse	8	0	cranioventral	1	more opaque	homogeneous	6	28.078	10.575	4	10	PEG
		1	cranioventral	1	less opaque	heterogeneous	2	4.834	11.106	2	4	
Fjord	12	0	cranioventral + caudoventral	1	more opaque	homogeneous	6	35.011	17.434	4	10	PEG
		1	cranioventral + caudoventral	1	more opaque	mixed	5	24.016	27.31	4	9	
Westphalian Horse	14	0	cranioventral + caudoventral	2	more opaque	mixed	5	14.868	35.047	4	9	PEG
		1	cranioventral + caudoventral	1	mixed	mixed	4	20.834	34.91	4	8	
Shetland Pony	7	0	Cranioventral + ventral	1	more opaque	homogeneous	6	33.276	12.797	4	10	PEG
		1	cranioventral	1	mix	mix	4	30.006	15.347	4	8	

Andalousian	8	0	Cranioventral + ventral	1	more opaque	homogeneous	6	35.002	19.473	4	10	SS
		1	Cranioventral + ventral	1	mix	mix	4	34.971	17.115	4	8	
Shetland Pony	7	0	cranioventral	1	more opaque	homogen	6	15.085	7.546	4	10	SS
		1	cranioventral	3	less opaque	heterogeneous	4	5.174	4.344	1	5	
Friesian	19	0	cranioventral	2	more opaque	homogen	6	32.257	9.636	4	10	PEG
		1					0			0	0	
KWPN	6	0	Cranioventral + ventral	2	more opaque	homogen	6	34.934	14.647	4	10	MO
		1	Cranioventral + ventral	2	mix	mix	4	34.982	14.674	4	8	
Warmblood	2	0	cranioventral	2	more opaque	mix	5	29.826	18.757	4	9	MO
		1					0			0	0	
Icelandic Horse	24	0	cranioventral	1	more opaque	homogen	6	21.586	7.92	4	10	PEG
		1	dorsale Colon	1	mix	mix	3	27.056	6.76	4	7	
Westphalian Horse	4	0	cranioventral	3	more opaque	homogen	8	32.632	10.524	4	12	SS
		1	cranioventral	2	mix	mix	6	28.35	9.904	4	10	
Fjord	21	0	cranioventral	1	more opaque	homogen	6	30.638	17.757	4	10	MO
		1	dorsale Colon	1	less opaque	heterogen	1	15.105	5.84	4	5	
Haflinger	23	0	cranioventral	1	more opaque	homo	6	11.746	25.621	4	10	MO
		1	cranioventral	1	less opaque	heterogen	2	2.503	10.101	3	5	
Thoroughbread	15	0	ventral	1	dichter	homogen	5	6.482	22.55	4	9	PEG
		1	medial	1	less opaque	heterogen	1	5.255	6.114	2	3	

Oldenburg	6	0	cranioventral	4	more opaque	homogen	8	13.805	34.957	4	12	PEG
		1	cranioventral	1	less opaque	heterogen	2	10.032	13.507	4	6	
German Riding Pony	4	0	cranial	1	mix	mix	3	13.535	14.464	4	7	SS
		1	ventral	1	mix	mix	3	0.6	5.382	1	4	
Irish Tinker		0	cranioventral	1	dichter	homogen	6	14.288	35.021	4	10	PEG
		1	cranioventral	1	mix	heterogen	3	4.221	11.916	3	6	
Friesian	12	0	cranioventral	1	more opaque	homogen	6	14.438	35.001	4	10	SS
		1	cranioventral	1	more opaque	homogen	6	9.908	31.961	4	10	
KWPN	16	0	cranioventral	1	more opaque	mix	5	17.385	35.047	4	9	PEG
		1	cranial	1	less opaque	heterogen	2	4.436	15.388	2	4	
Haflinger	1	0	cranioventral	2	mixed	mixed	4	21.931	11.484	4	8	SS
		1	cranioventral	2	mixed	mix	5	32.944	14.42	4	8	
